# Mitral Valve Replacement in a Young Pregnant Woman: A Case Report and Review of Literature

**DOI:** 10.5812/cardiovascmed.17561

**Published:** 2014-04-01

**Authors:** Mhonchan Kikon, Krishnanu Dutta Choudhury, Neeraj Prakash, Anubhav Gupta, Vijay Grover, Vijay Kumar Gupta

**Affiliations:** 1 Department of Cardiothoracic and Vascular Surgery, Post Graduate Institute of Medical Education and Research, Dr. Ram Manohar Lohia Hospital, New Delhi, India

**Keywords:** Mitral Valve, Replacement, Pregnancy

## Abstract

**Introduction:**

Cardiac diseases occur in 2-4% of pregnancies and rheumatic mitral disease is the most common acquired heart disease in pregnancy. Cardiac surgery carries significant maternal and fetal complications. Cardiac operation during pregnancy is indicated only when medical management fails. Although emergency cardiac surgery during pregnancy increases fetal mortality, sometime urgent cardiac surgery is inevitable. Cardiac surgery can be performed with relative safety during pregnancy by adopting normothermic, high flow rate circulation and continuous fetal activity monitoring.

**Case Presentation:**

We reviewed English literature of a pregnant patient undergoing cardiac surgery during pregnancy. We presented a 25-year-old woman admitted with massive hemoptysis.

**Discussion:**

The patient underwent a successful mitral valve replacement during the third trimester. The aim of our study was to propose a practical guideline for similar situations.

## 1. Introduction

Cardiac diseases occur in 2-4% of pregnancies and rheumatic mitral disease is the most common acquired heart disease in pregnancy (1). Mitral stenosis is the most common valvular pathology. Although maternal mortality remains the same as that of the non-pregnant women, fetal mortality rate still remains high due to using cardiopulmonary bypass. Herein, we presented a successful mitral valve replacement in a 25-year-old pregnant woman at third trimester admitted with massive hemoptysis.

## 2. Case Presentation

A 25-year-old woman, gravid 1, para 0, at 28 weeks of gestation presented with a complaint of massive hemoptysis, palpitation (NYHA III-IV) and dyspnea (NYHA-IV). On physical examination, the patient blood pressure was 88/64 mmHg, and pulse rate was 98/min with regular pulse rate. Her hemoglobin was 8 mg/dL. Chest X-ray showed cardiomegaly with pulmonary plethora. Electrocardiography of the patient showed normal sinus rhythm with normal axis. Transthoracic echocardiography demonstrated rheumatic heart disease, severe calcific mitral stenosis (MVA ~ 0.9 cm^2^, Mean gradient ~ 33 mmHg), severe mitral regurgitation (vena contracta ~ 0.7 cm), severe tricuspid regurgitation with ejection fraction of 40-45% and severe pulmonary artery hypertension, left atrial size of approximately 7.4 cm, and dilated left ventricle (end systolic dimension ~ 50 mm). Chest HRCT (High Resolution Computed Tomography) was performed with fetal guard to rule out any lung lesions before preparing the patient for surgery, as her hemoptysis was not controlled with medical therapy. HRCT revealed normal results. On admission, patient was initially managed with beta-blocker (Atenolol), furosemide, spironolactone and other conservative measures like complete bed rest, intensive monitoring of hemodynamics and fetal heart beat. Despite all conservative measures, the patient status was deteriorated. An emergency surgery was undertaken with an increased risk to the fetus’s life, because the mother's life was at risk. After informed consent was obtained, mitral valve replacement with 31 mm St. Jude Medical mechanical heart valve prosthesis (St. Jude Medical Inc., St. Paul, Minnesota, USA) was performed. Fetal cardiac activity was monitored with an external ultrasound transducer. Normothermic, nonpulsatile CPB (cardiopulmonary bypass) was performed using a high flow rate of greater than 2.5 L/m^2^/min. Flow rate was adjusted to maintained mean arterial pressure greater than 70 mmHg. Prior to initiating Cardiopulmonary bypass (CPB) full heparinization (3 mg/kg) was provided to keep the ACT (activated clotting time) above 450 seconds. The aortic cross clamp and CPB times were 34 minutes and 1 hour 3 minutes respectively. No fetal distress was noted. Fetal movements were normal postoperatively. Immediate postoperative period, heparin was administered followed by oral anticoagulation (warfarin) after extubation. The patient had uneventful postoperative course and was discharged from the hospital on fifth postoperative day. The woman gave birth to a full-term baby at 38 weeks of gestation by cesarean section. Until the last visit (eleven month after surgery), both the patient and child were well. The patient was followed up for one and half years, she was pregnant with the second child, and was asymptomatic.

## 3. Discussion

CPB during pregnancy is associated with a maternal mortality rate similar to that of the nonpregnant female (3-15%). However, fetal mortality still remains high at about 9-30% (2-4). Due to increased maternal and fetal complication and high fetal mortality rate, valvular repair or replacement during pregnancy is indicated in selected patients who remain symptomatic despite adequate medical therapy (5). In our case the patient was refractory to medical management and was found to have a high-risk pregnancy, once she was in NYHA class III-IV with severe anemia (hemoglobin ~ 8 mg/dL) and valvular lesions, such as severe mitral stenosis, severe mitral regurgitation and a severe pulmonary artery hypertension.

During pregnancy, significant cardiovascular changes occur and increase the cardiac workload. Cardiac output increases 40-50% above the resting levels secondary to rise in intravascular volume. Hemodilution, which maintains maternal hematocrit at about 28% is important during CPB. Oxygen consumption rises to 15% above the non-pregnant levels. High flow rate circulation (20% to 40% higher than nonpregnant patients) maintaining mean perfusion pressure greater than 2.4 L/m^2^/min is recommended by various authors. Pump flow should be sufficient to maintain a mean arterial pressure above 70 mmHg. IABP (intra-aortic balloon pump) has been used during bypass for pulsatile perfusion to improve the fetal outcome (6).

During CPB, maintaining normothermia plays an important role in improving the fetal outcome. Earlier studies confirmed that hypothermia (temperature below 35^o^C) results in higher fetal mortality. During hypothermia, gas exchange is decreased at the placental level due to increased uterine tone and contraction. Uterine contractions are particularly common during the rewarming phase after moderate or profound hypothermia. Avoiding hypothermia can prevent contractions. In our case, temperature was maintained above 35°C during the bypass period.

Gestational age at the time of surgery is considered as a contributing risk factor for fetal morbid-mortality (1). Early intervention would decrease maternal risk but may result in fetal loss. Second trimester is reported to be the ideal age of gestation for good fetal outcome as there is higher risk of fetal malformations in the first trimester and preterm delivery in the third (1, 7). There is a decline in fetal mortality rate with delayed cardiac surgery as reported by Weiss and colleagues (4).

Fetal and uterine activity monitoring can be useful during CPB and has been reported to reduce fetal mortality rate to 9.5% (8). External ultrasonography and cardiotocography are used for fetal heart beat monitoring and uterine activity respectively. Bradycardia is the most common fetal response to CPB, most likely because of hypoperfusion. CPB is a strong stimulus for uterine contraction, their frequency increases with increasing gestational age. Uterine contractions are associated with significant fetal loss. Fetal bradycardia and increased uterine contraction require immediate treatment. Indomethacin suppository or magnesium administration may be required for prevention of uterine contraction. Terbutaline and ritodrine are also effective agents.

High potassium level in the maternal blood secondary to cardioplegia infusion affects the fetal heat leading to bradycardia and cardiac arrest. Cardioplegic effluent should be aspirated from the right atrium to avoid mixing with the venous drainage. Biological tissue valves do not require anticoagulation, and are usually considered the most suitable devices in women of childbearing age. However, unlike mechanical heart valves they are prone to rapid degeneration in early period. Mechanical valves are preferred for their durability but the need for anticoagulation makes it undesirable in these patients. In our case, mechanical prosthetic valve (MPV) was offered to patient, as the patient was in favor of MPV for its durability. Patient was explained about the lifelong oral anticoagulation. Patient was received warfarin long-life maintaining an INR (International Normalized Ratio) of about 2.5-3.5.

During pregnancy, platelet number and activation of coagulation cascades increase, in contrast fibrinolytic activity decreases. Managing of anticoagulation in a pregnant woman is very important as pregnancy increases the risk of mechanical prosthetic heart valve (MPHV) thrombosis. It is reported to be between 0.7% and 6% per patient per year (up to 25% in the absence of adequate anticoagulation) (9). Given the high teratogenic risk of oral anticoagulant (warfarin), low molecular weight heparin (LMWH) is the first choice during the first trimester pregnancy with MPHV. The guideline recommends evaluating anti-Xa when administering LMWH to a pregnant woman. LMHW is found to be less effective even with adequate range of anti Xa levels, and unfractionated heparin or LMWH has been found to have a higher risk of developing thromboembolic complications compared to warfarin. Warfarin is recommended up to the 36th week of pregnancy except for the period between sixth to 12th weeks. Warfarin is found to be the most effective drug for the prevention of MPHV thrombosis. 

Use of CPB has been previously shown to be associated with relatively high fetal mortality, although maternal mortality rate is similar to that of the nonpregnant female. Surgery with CPB during the third trimester pregnancy is associated with higher risk of complication with high chance of fetal loss. However, a successful strategy for fetal survival can be adopted during emergent surgery when medical management fails in pregnant patients. A practical guideline include:

1. high flow rate circulation (> 2.5 L/min/m^2^), and use of pulsatile perfusion (IABP) to maintain mean perfusion pressure more than 70 mmHg;
2. normothermic CPB and avoiding deep hypothermia to minimize rewarming;
3. continuous fetal and uterine activity monitoring for early recognition of potential problems;
4. avoiding cardiac surgery during the first 24 weeks of gestation;
5. scavenging of cardioplegic effluent from the right atrium to prevent mixing with the perfusate.

More investigations are recommended on this issue due to the high fetal mortality associated with cardiopulmonary bypass.
